# Supplementary material for: fruit-SALAD: A Style Aligned Artwork Dataset to reveal similarity perception in image embeddings
Source: Sci Data. 2025 Feb 12;12:254. doi: 10.1038/s41597-025-04529-4 (PMC11821872; doi:10.1038/s41597-025-04529-4)
Supplement: Supplementary file 1 — Supplementary Information [file 41597_2025_4529_MOESM1_ESM.pdf]

Supplementary information

Figure S1 2

Figure S2 3

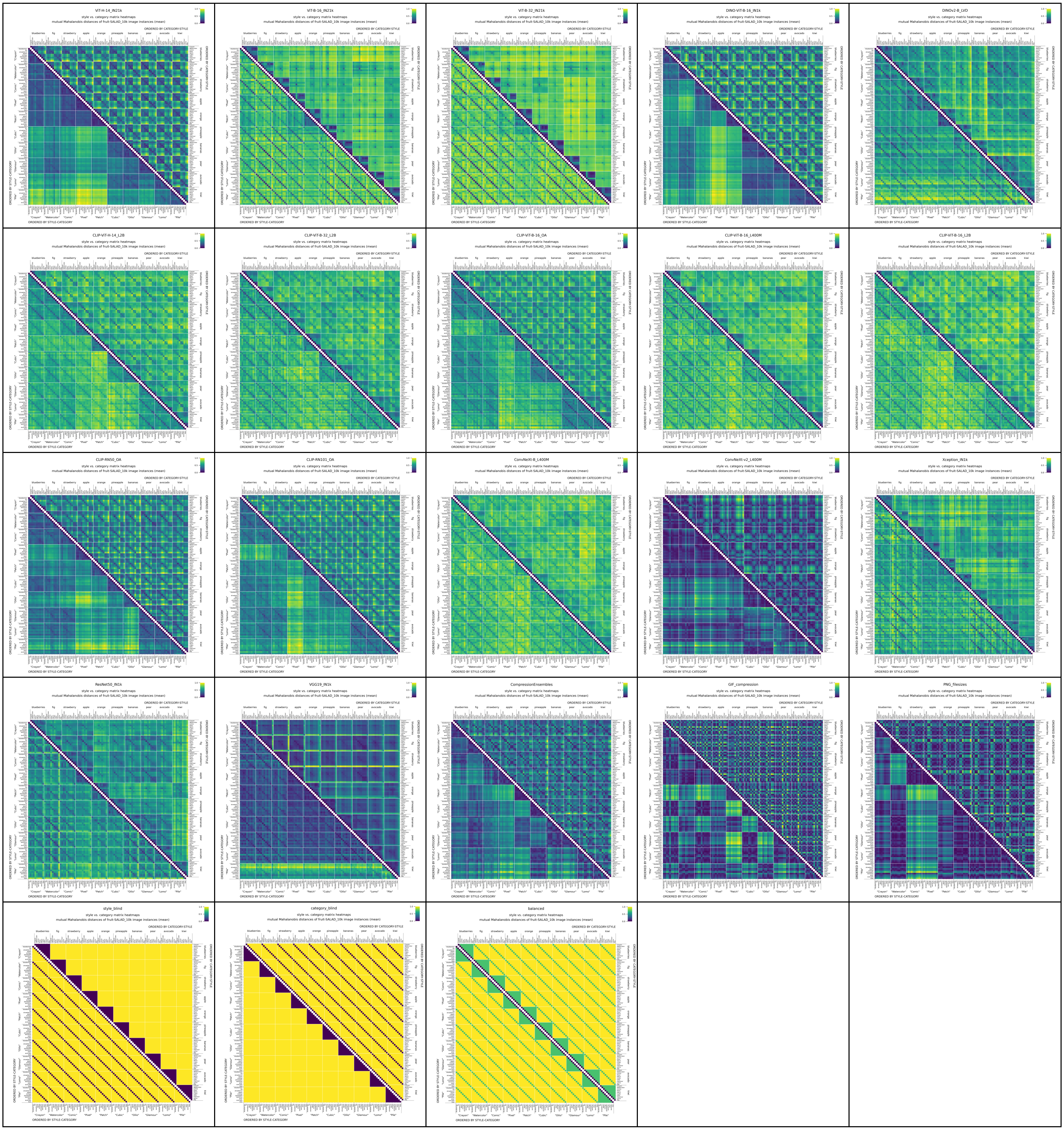

Figure S1. Model heatmaps indicating the mutual Mahalanobis distance of fruit-SALAD images. The matrix cells correspond to the mean of all 10,000 distance pairs of 100 by 100 instances of fruit-SALAD images. Below the diagonal: sorted by style first and fruit category second. Above the diagonal: sorted by fruit category first and style second. The color indicates the pairwise Mahalanobis distance of image embedding vectors obtained from the respective model or algorithm, from low to high (blue to yellow) while low values indicate higher similarity. The figure construction is comprehensive as the matrices are symmetric; diagonal cells can be left out.

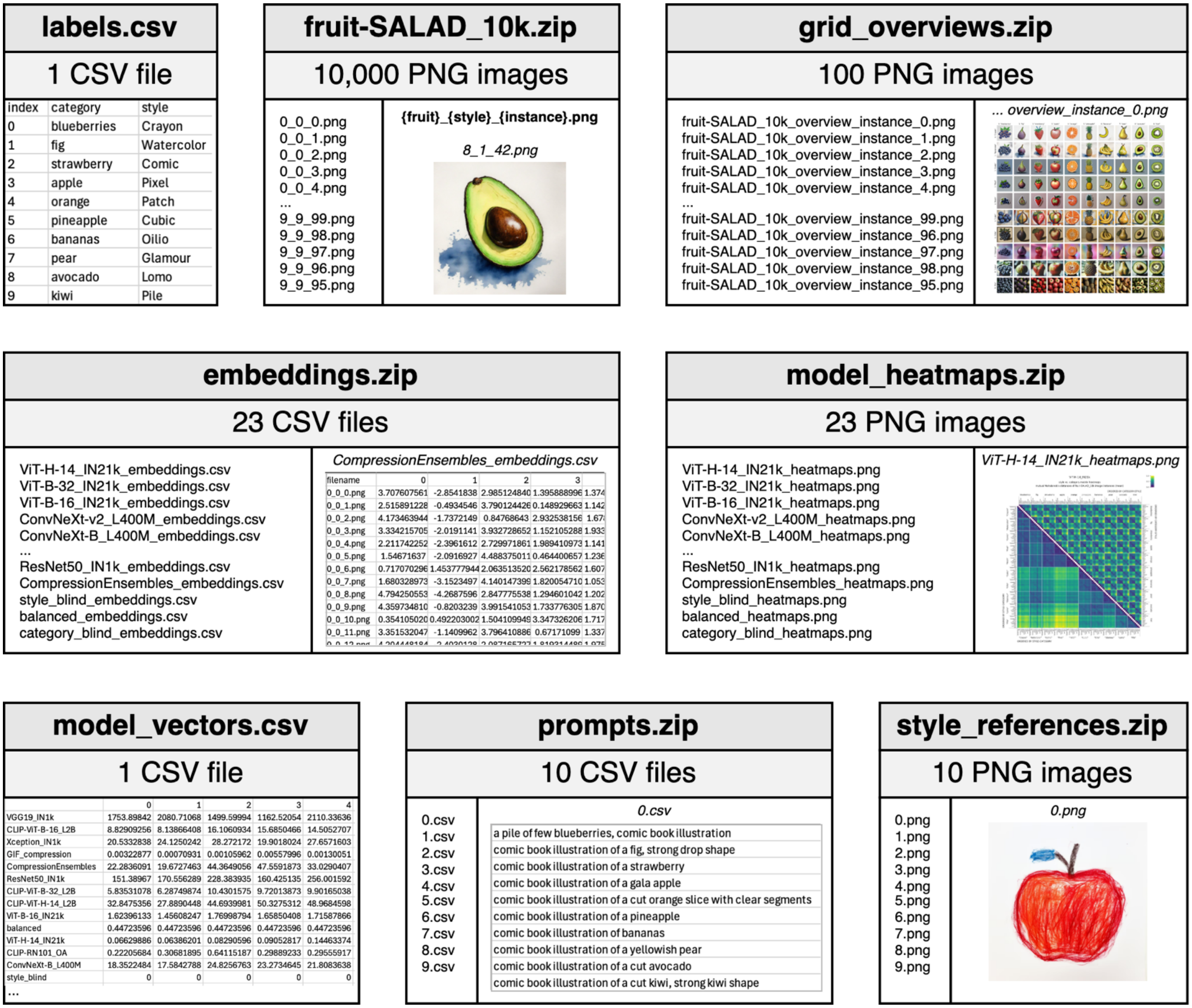

Figure S2. Overview of all dataset repository files.
